# Example dataset for the hMRI toolbox

**DOI:** 10.1016/j.dib.2019.104132

**Published:** 2019-06-11

**Authors:** Martina F. Callaghan, Antoine Lutti, John Ashburner, Evelyne Balteau, Nadège Corbin, Bogdan Draganski, Gunther Helms, Ferath Kherif, Tobias Leutritz, Siawoosh Mohammadi, Christophe Phillips, Enrico Reimer, Lars Ruthotto, Maryam Seif, Karsten Tabelow, Gabriel Ziegler, Nikolaus Weiskopf

**Affiliations:** a Wellcome Centre for Human Neuroimaging, London, UK; b Laboratory for Research in Neuroimaging, Department of Clinical Neuroscience, Lausanne University Hospital and University of Lausanne, Switzerland; c GIGA Institute, University of Liège, Liège, Belgium; d Department of Neurology, Max Planck Institute for Human Cognitive and Brain Sciences, Leipzig, Germany; e Medical Radiation Physics, Department of Clinical Sciences Lund, Lund University, Lund, Sweden; f Department of Neurophysics, Max Planck Institute for Human Cognitive and Brain Sciences, Leipzig, Germany; g Medical Center Hamburg-Eppendorf, Hamburg, Germany; h Emory University, Atlanta, USA; i Spinal Cord Injury Centre, University Hospital Balgrist, University of Zurich, Zurich Switzerland; j WIAS Berlin, Germany; k Institute for Cognitive Neurology and Dementia Research, University of Magdeburg, Germany

**Keywords:** hMRI, hMRI toolbox, qMRI, Quantitative MRI, Analysis tools, qMRI software

## Abstract

The hMRI toolbox is an open-source toolbox for the calculation of quantitative MRI parameter maps from a series of weighted imaging data, and optionally additional calibration data. The multi-parameter mapping (MPM) protocol, incorporating calibration data to correct for spatial variation in the scanner's transmit and receive fields, is the most complete protocol that can be handled by the toolbox. Here we present a dataset acquired with such a full MPM protocol, which is made freely available to be used as a tutorial by following instructions provided on the associated toolbox wiki pages, which can be found at http://hMRI.info, and following the theory described in: hMRI – A toolbox for quantitative MRI in neuroscience and clinical research [1].

**Table:** Specifications Table

Subject area	Neuroimaging
More specific subject area	Quantitative MRI, Multi-Parameter Mapping (MPM)
Type of data	*In vivo* MRI data (Fig. 1, Fig. 2, Table 1)
How data was acquired	3T MRI, Siemens Prisma
Data format	NIfTI image volumes after defacing for anonymization (only)
Experimental factors	A deliberate motion was performed during the experiment after acquisition 9 and returned to approximately the original position after acquisition 12.
Experimental features	No special treatment was performed except to anonymise via SPM∖Util∖De-face Images.
Data source location	Wellcome Centre for Human Neuroimaging, London, UK
Data accessibility	Data is available at http://hMRI.info
Related research article	Tabelow K, Balteau E, Ashburner J, Callaghan MF, Draganski B, Helms G, Kherif F, Leutritz T, Lutti A, Phillips C, Reimer E, Ruthotto L, Seif M, Weiskopf N, Ziegler G, Mohammadi S (2019). hMRI - A toolbox for quantitative MRI in neuroscience and clinical research. NeuroImage, in press [1].

**Table:** 

**Value of the data** • These data can be used as an educational tool for use in conjunction with the hMRI toolbox [1]. • These data can be used to develop and test novel algorithms for estimating quantitative MRI (qMRI) parameters in the human brain.

## 1 Data

This dataset is comprised of imaging volumes (full list in Table 1) acquired with the multi-parameter mapping quantitative MRI protocol. It consists of calibration data to map the transmit field (series 4), main B_0_ field (series 5 and 6) and the net receive field (series 7, 8, 10, 11, 13 and 14). It also consists of multi-echo volumes with variable flip angle (series 9 and 15) and additional magnetisation transfer (MT) weighting (series 12).

A tutorial describing how to process the dataset with the hMRI toolbox is available at https://github.com/hMRI-toolbox/wiki/MapCreation#example. From these data maps of the effective transverse relaxation rate (R_2_*), the longitudinal relaxation rate (R_1_), proton density (PD) and magnetisation transfer saturation (MT) can be generated.

The acquisition of these data was approved by the local ethics committee and informed written consent was obtained from the participant prior to scanning. All data were acquired on a whole body 3T Prisma system (Siemens Healthineers, Erlangen, Germany). The data were acquired using the body coil for signal transmission and a 64 channel coil for signal reception.

## 2 Experimental design, materials, and methods

A summary of the data acquisition and experimental design is given in Table 1. The participant was centred within the head coil at the outset of the exam.

**Table 1:** Image series number, showing the chronology of the acquisition, together with the sequence name and the data description.

Image Series No.	Sequence Name	Description
4	mfc_seste_b1map_v1e	B_1_^+^ Mapping Data
5	gre_field_mapping_1acq_rl	B_0_ Mapping Magnitude
6	gre_field_mapping_1acq_rl	B_0_ Mapping Phase Difference
7	mfc_smaps_v1a_Array	Net Receive Sensitivity Mapping of Array
8	mfc_smaps_v1a_QBC	Net Receive Sensitivity Mapping of Body Coil
9	pdw_mfc_3dflash_v1i_R4	Lower flip angle multi-echo FLASH
*Participant moved to new position via primary rotation about z*		
10	mfc_smaps_v1a_Array	Net Receive Sensitivity Mapping of Array
11	mfc_smaps_v1a_QBC	Net Receive Sensitivity Mapping of Body Coil
12	mtw_mfc_3dflash_v1i_R4	FLASH acquisition with MT pre-pulse
*Participant returned to approximate alignment with the original position*		
13	mfc_smaps_v1a_Array	Net Receive Sensitivity Mapping of Array
14	mfc_smaps_v1a_QBC	Net Receive Sensitivity Mapping of Body Coil
15	t1w_mfc_3dflash_v1i_R4	Higher flip angle multi-echo FLASH

### 2.1 B_1_^+^ mapping data (image series No.4)

**Fig. 1:**
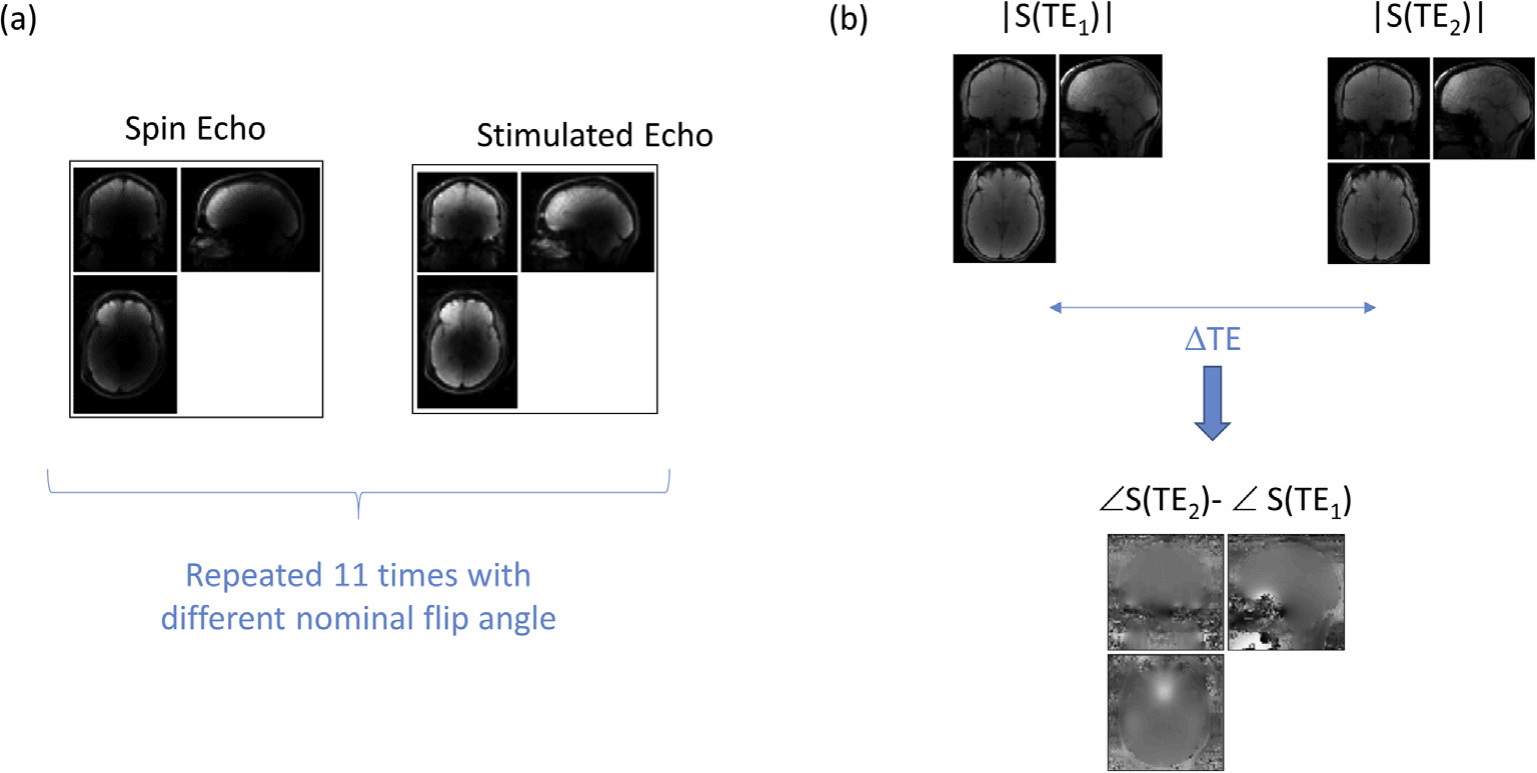
Calibration data to map the transmit field, B_1_^+^. Eleven spin echo and stimulated echo pairs were acquired (for robustness to low SNR regions) with different nominal flip angle (a). These data were acquired with an EPI readout. To correct for resulting geometric image distortions, additional calibration data mapping spatial inhomogeneity in the main magnetic field were also acquired (b).

The scanning session began by acquiring calibration data (Fig. 1a, mfc_seste_b1map_v1e_004) to measure B_1_^+^ following a previously published method [2], [3]. Eleven spin-echo and stimulated-echo pairs were acquired with the nominal flip angle (i.e. α in an α-2α-α sequence) varying from 115° to 65° in 5° decrements (Fig. 1a). These data were acquired with 4mm isotropic resolution using a 3D-EPI readout with a 0.5 ms echo spacing. The sequence had an echo time of 39.06 ms, a mixing time of 33.8 ms and a repetition time (TR) of 500 ms. The field of view (FoV) was 256 (anterior-posterior, AP) x 192 (right-left, RL) x 192 (head-foot, HF) mm^3^. Partially parallel imaging with a speed up factor of 2 was used in each phase-encoded direction. A fully sampled volume was acquired at the outset to serve as the auto-calibrating lines for subsequent reconstruction of the aliased data using the GRAPPA algorithm [4] as implemented in the vendor's software. The primary phase-encoded direction of the EPI readout was right-left. The total acquisition time was 3 minutes.

### 2.2 B_0_ mapping (image series No.5–6)

To correct for geometric distortions in the B_1_^+^ mapping data caused by the low bandwidth in the phase-encoded direction of the EPI readout (RL), additional data were acquired to map the inhomogeneity of the B_0_ field (gre_field_mapping_1acq_rl) and subsequently used to apply distortion correction on the B_1_^+^ calibration data. These data were acquired with 3mm effective isotropic resolution using a multi-echo gradient echo sequence with an excitation flip angle of 90°, a TR of 1.02s and a bandwidth of 260 Hz/pixel. Magnitude images with echo times of 10.00 and 12.46 ms respectively (gre_field_mapping_1acq_rl_0005) were reconstructed together with their phase difference (gre_field_mapping_1acq_rl_0006) by the vendor's software (Fig. 1b). The acquisition time was 2 minutes 14 seconds.

### 2.3 FLASH acquisitions (image series No 9, 12 and 15)

**Fig. 2:**
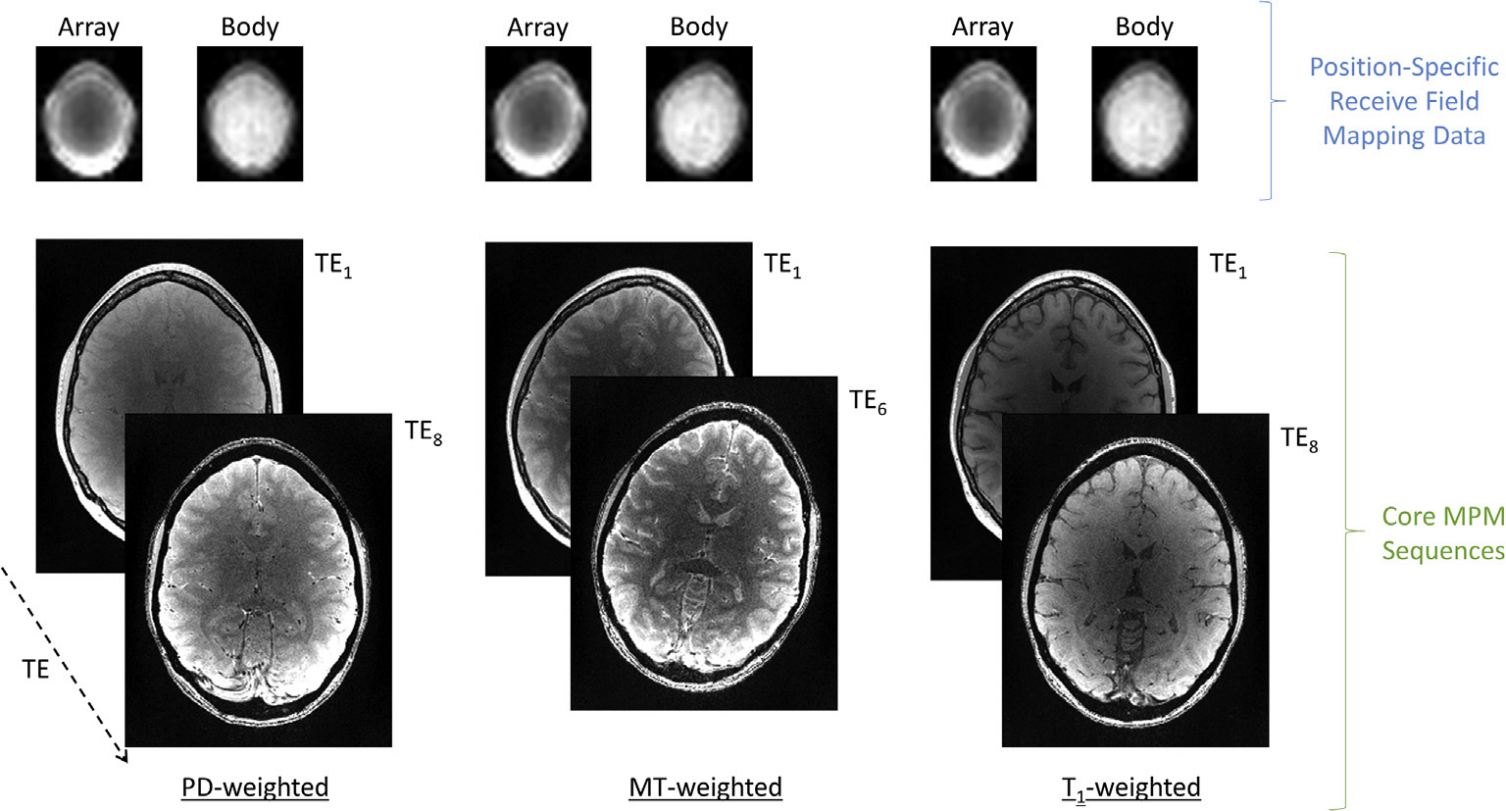
The main MPM protocol consists of three high resolution, multi-echo, 3D FLASH volumes with proton density (PD), magnetisation transfer (MT) and T_1_ weighting. Prior to each high resolution acquisition shorter, low resolution, single echo acquisitions were acquired to map the net receive field sensitivity of the array coil, which will vary if inter-scan motion occurs (c.f. PD-weighted v's MT-weighted).

These were followed by the acquisition of spoiled multi-echo 3D fast low angle shot (FLASH) acquisitions with predominantly PD, MT or T_1_ weighting (Fig. 2, pdw_mfc_3dflash_v1i_R4_0009, mtw_mfc_3dflash_v1i_R4_0012 and t1w_mfc_3dflash_v1i_R4_0015 respectively). Each multi-echo FLASH volume had a TR of 25 ms.

The PD-weighting was achieved with an excitation flip angle of 6°. The flip angle was increased to 21° to achieve T_1_-weighting. MT-weighting was achieved by applying a Gaussian RF pulse 2 kHz off resonance prior to excitation with a flip angle of 6°. The off-resonance MT saturation pulse was 4 ms in duration and had a nominal flip angle of 220°.

Each of these volumes were acquired with whole-brain coverage using a FoV of 256 (HF) x 224 (AP) x 179 (RL) mm^3^. Gradient echoes were acquired with alternating readout gradient polarity at eight equidistant echo times ranging from 2.30 to 18.40 ms in steps of 2.30 ms using a readout bandwidth of 488Hz/pixel. Only six echoes were acquired for the MT-weighted acquisition in order to maintain a TR of 25 ms for all FLASH volumes. To maximise spoiling of the transverse magnetisation, each FLASH volume was acquired with RF spoiling using a linear phase increment of 137°. In addition, a spoiling gradient moment, which imposed a 6π dephasing moment across a voxel dimension, was applied along the readout direction after the last echo had been acquired. To accelerate the data acquisition, partially parallel imaging was employed in each phase-encoded direction (AP and RL) with a speed up factor of 2 and forty integrated auto-calibrating lines in each direction for subsequent reconstruction with GRAPPA [4]. The acquisition time for each FLASH volume was 7 minutes 8 seconds. Each dataset was acquired with a 30° rotation of the sagittal plane so that any eye-related motion artefact propagated to the neck/inferior cerebellum rather than the cortex.

### 2.4 Net receive sensitivity mapping of array (image series no. 7, 10 and 13) and body coil (image series no. 8, 11 and 14)

Prior to the acquisition of each FLASH volume described above, two additional unaccelerated, low resolution (8 mm isotropic) volumes were acquired with the same FoV (Fig. 2). A single echo, with a TE of 2.20 ms, was acquired in each case using a 6° flip angle and a TR of 6.00 ms. The acquisition time for each of these calibration volumes was 5.90 seconds. The first was obtained using the 64 channel coil for signal reception (mfc_smaps_v1a_Array*), while the second was acquired using the body coil for signal reception (mfc_smaps_v1a_QBC*). These data were acquired to correct for the relative receive field sensitivity of the array coil, which will be position-specific [5].

### 2.5 Deliberate inter-scan motion

After the PD-weighted acquisition had been acquired, the participant performed a yaw rotation (i.e. about the z-axis) while the scanner was not running (see Table 1). The amplitude of the motion aimed to be as large as possible within the confines of the 64 channel coil.

This was done to be able to test the performance of the inter-scan motion correction scheme that accounts for position-specific modulation by the receiving coil. After the participant had moved sensitivity mapping data (image series 10–11) were acquired followed by acquisition of the MT-weighted dataset (image series 12).

The participant then returned to approximately the original position (i.e. that of the PD-weighted acquisition, centred within the head coil), again while the scanner was not running. Then, sensitivity mapping data (image series 13–14) were acquired followed by acquisition of the T1-weighted dataset (image series 15).

### 2.6 Processing

Prior to sharing, the DICOM images produced by the scanner were converted to NIfTI format using the DICOMImport utility as implemented in the hMRI toolbox ([1], http://hMRI.info). The NIfTI data were subsequently anonymised using the defacing utility as implemented in SPM12 (https://www.fil.ion.ucl.ac.uk/spm/) in order to comply with General Data Protection Regulation (GDPR) regulations.

**Fig. 3:**
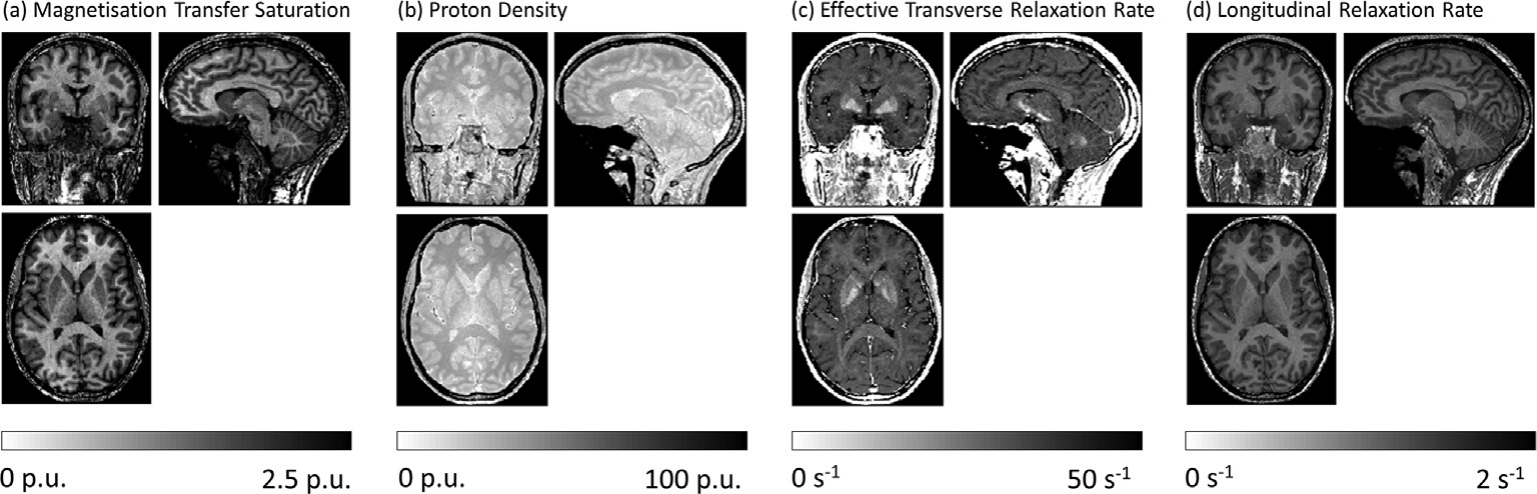
Orthogonal views of the quantitative multi-parameter maps magnetisation transfer saturation (a), proton density (b), effective transverse relaxation rate (c) and longitudinal relaxation rate (d), derived from this dataset using the hMRI toolbox.

Following anonymization, the data were processed using the “Create hMRI maps” module of the hMRI toolbox. This module is accessed via the SPM batch menu via: SPM-Tools-hMRI Tools-Create hMRI maps. The processing included correction for receive field modulation as described in Papp et al. [5], transmit field inhomogeneity [2], [3] and imperfect RF spoiling correction [6]. The batch module, together with the toolbox configuration file are also supplied. Orthogonal views of the resulting maps are shown in Fig. 3. All outputs from this module, including the supplementary results (B_1_^+^ and B_1_^−^ maps, and signal intensities for each contrast extrapolated to TE = 0 ms) and all meta-data (processing log, json files, and quality assurance (QA) metrics), are provided.
